# Identification of SHMT2 as a Potential Prognostic Biomarker and Correlating with Immune Infiltrates in Lung Adenocarcinoma

**DOI:** 10.1155/2021/6647122

**Published:** 2021-04-08

**Authors:** Lianxiang Luo, Yushi Zheng, Zhiping Lin, Xiaodi Li, Xiaoling Li, Mingyue Li, Liao Cui, Hui Luo

**Affiliations:** ^1^The Marine Biomedical Research Institute, Guangdong Medical University, Zhanjiang 524023, China; ^2^The Marine Biomedical Research Institute of Guangdong Zhanjiang, Zhanjiang, Guangdong 524023, China; ^3^The First Clinical College, Guangdong Medical University, Zhanjiang 524023, China; ^4^The Orthopedic Department, The Affiliated Hospital of Guangdong Medical University, Zhanjiang 524023, China; ^5^Animal Experiment Center, Guangdong Medical University, Zhanjiang 524023, China; ^6^Department of Pathology and Laboratory Medicine, Perelman School of Medicine, University of Pennsylvania, Philadelphia, PA19104, USA; ^7^Guangdong Key Laboratory for Research and Development of Natural Drugs, Guangdong Medical University, Zhanjiang, Guangdong 524023, China

## Abstract

It has attracted growing attention that the role of serine hydroxy methyl transferase 2 (SHMT2) in various types of cancers. However, the prognostic role of SHMT2 in lung adenocarcinoma (LUAD) and its relationship with immune cell infiltration is not clear. In this study, the information of mRNA expression and clinic data in LUAD were, respectively, downloaded from the GEO and TCGA database. We conducted a biological analysis to select the signature gene SHMT2. Online databases including Oncomine, GEPIA, TISIDB, TIMER, and HPA were applied to analyze the characterization of SHMT2 expression, prognosis, and the correlation with immune infiltration in LUAD. The mRNA expression and protein expression of SHMT2 in LUAD tissues were higher than in normal tissue. A Kaplan-Meier analysis showed that patients with lower expression level of SHMT2 had a better overall survival rate. Multivariate analysis and the Cox proportional hazard regression model revealed that SHMT2 expression was an independent prognostic factor in patients with LUAD. Meanwhile, the gene SHMT2 was highly associated with tumor-infiltrating lymphocytes in LUAD. These results suggest that the SHMT2 gene is a promising candidate as a potential prognostic biomarker and highly associated with different types of immune cell infiltration in LUAD.

## 1. Introduction

Lung cancer is the most common cancer and the main reason of cancer-related death, leading to a rising public concern worldwide. Lung cancer is divided into nonsmall cell lung cancer (NSCLC) and small cell lung cancer (SCLC). NSCLC accounts for approximately 85% of all lung cancers [1], which contain two main types: lung squamous cell carcinoma (LUSC) and lung adenocarcinoma (LUAD). LUAD is the most common histological subtype of NSCLC diagnosed, followed by LUSC. As the most common histological subtype, LUAD frequently occurs in females and nonsmoking people, with no obvious clinical symptoms in the early stage, but shared some common symptoms with other respiratory diseases, resulting in difficulty in identification of lung cancer. In addition, LUAD has an average 5-year survival rate of less than 20% [2] due to its metastasis at early stages. Therefore, there is an urgent need to identify new diagnostic and prognostic biomarkers for LUAD to increase the efficacy of early diagnosis.

Serine hydroxy methyl transferase (SHMT) is an essential enzyme in the conversion between serine and glycine as well as one-carbon metabolism, providing the important precursors for protein and nucleic acid synthesis for cancer growth and metastasis. To be noted, amino acid and one-carbon metabolism are the basis of cancer biology, and hyperactivation of one-carbon metabolism has been proved to be driving factors of cell proliferation and related to the epigenetic state of the cell [3]. SHMT2 is one of SHMT genes, encodes a protein that localizes to the mitochondria [4], and is identified as a potential driving gene in diverse cancers in cell growth and aggressiveness [5]. As a key regulator of viral transcription, HIV-1 Tat levels are regulated through K63Ub-selective autophagy-mediated through SHMT1,2 and the BRCC36 deubiquitinase. Xu et al. has identified SHMT2 and BRCC36 as novel and important regulators of HIV-1 Tat protein levels in infected T cells [6]. Ji et al. proved expression levels of SHMT2 in HCC tissues were significantly correlated with tumor grade and hepatitis B virus (HBV) infection [7]. Besides, genetic ablation of SHMT2 causes strong increases in inflammatory cytokine signatures [8]. SHMT2 may alleviate the apoptosis and the release of damaging inflammatory factors after hepatic ischemia-reperfusion injury by inhibiting the activation of the JNK pathway and excessive activation of the NF-*κ*B pathway [9]. SHMT2 showed unfavorable overall survival to intrahepatic cholangiocarcinoma patients [10]. SHMT2 is a very crucial gene in many cancers, and proteomic profiling of breast cancer metabolism identifies SHMT2 as a prognostic factor [11], and it drives glioma cell survival in ischemia depending on glycine clearance [12, 13]. However, the immune-related SHMT2 in LUAD and its potential use in prognosis are still largely unknown.

In recent years, the combination of immunotherapy and high-throughput gene microarray has been widely employed for oncology and other disease areas to analyze deeper correlation to predict more insight for research. So, analysis of available high-throughput data in many databases has become an effective and low-cost method to discover biomarkers for many diseases. Immune cells have an intimate connection with the prognosis in various cancers. Mounting evidence supports that the malignant phenotype is not only determined by the intrinsic activities of cancer cells but also by components in the tumor microenvironment, especially tumor-infiltrating immune cells [14], which is an important determinant of prognosis and immunotherapy response of lung cancer [15]. For example, CD83 + dendritic cells and Foxp3 + regulatory T cells in primary lesions and regional lymph nodes are negatively correlated with the prognosis of gastric cancer [16]. Increased tumor-infiltrating tumor-associated macrophages (TAMs) are associated with a poor prognosis of NSCLC [17]; DC and T cells are connected with better prognosis [18, 19]. Meanwhile, high-throughput gene microarray makes it accessible for us to further explore the tumors at multiple levels.

In this study, we downloaded the LUAD-related data sets from the GEO database (Gene Expression Omnibus) and TCGA (The Cancer Genome Atlas) database and conducted bioinformatics statistical analysis to select different expression genes (DEGs) between normal tissue and tumor tissue. Subsequently, functional analysis and survival analysis were subsequently carried out to select and verify signature genes with biological and clinical signatures. In addition, we took full advantage of convenient online site tools to explore the relationship between signature and immune cells and verify the suppose at multiple levels especially.

## 2. Methods

### 2.1. Data Collection and Preprocess

We obtained the LUAD-related microarray profiles (GSE116959 [20], GSE21933 [21], and GSE31210 [22]) from the GEO database (https://www.ncbi.nlm.nih.gov/geo/). In this study, the datasets that met the following criteria were selected: (a) studies of comparing gene expression between human LUAD cancer samples and corresponding normal tissues; (b) the number of samples in each gene expression profiling dataset should be more than 30.

The microarray data were normalized and analyzed via the R “limma” package, which implements empirical Bayesian methods for analyzing microarray data [23]. We set log2 fold change (FC) ≥ 1 with an adjusted *P* value less than 0.01 as the threshold to define important differentially expressed genes (DEGs) which are selected for subsequent analysis. We named the DEGs that overlapped in the three data matrixes as common DEGs. In addition, multiple probes corresponded to the same gene in the annotation file; the average expression of these probes was used as the expression value of the corresponding gene. Analyzing and processing these abovementioned data by R language.

Furthermore, we obtained the LUAD transcriptome RNA-seq data set and corresponding clinical data set from the TCGA database (https://cancergenome.nih.gov/) containing 521 tumor samples and 46 normal samples.

### 2.2. Functional Enrichment Analysis of DEGs

#### 2.2.1. GO and KEGG Pathway Analysis

In order to investigate biological processes functions and pathways associated with the selected DEGs, we also performed Gene Ontology (GO) and the Kyoto Encyclopedia of Genes and Genomes (KEGG) enrichment analyses. The GO analyses classified the common DEGs into three categories, including biological process (BP), cellular component (CC), and molecular function (MF). The KEGG analysis was conducted to determine significantly enriched the pathways of DEGs which was defined as the cutoff significant criteria with *P* value < 0.05. Besides, the Cytoscape software (version 3.8.0) was used to screen hub genes. The GO and KEGG analyses were both based on the online database DAVID (version 6.8) (https://david.ncifcrf.gov) and visually display through R software (version 3.6.1).

#### 2.2.2. Screening Hub Genes by Cytoscape Software

Cytoscape software (version 3.8.0) is an open-source bioinformatic software platform for visualizing molecular interaction networks and biological pathways and integrating these networks with annotations, gene expression profiles, and other state data. MCODE is a Cytoscape APP that finds clusters (highly connected areas) in the network.

#### 2.2.3. Gene Set Enrichment Analysis

Gene Set Enrichment Analysis (GSEA) (http://software.broadinstitute.org/gsea/index.jsp) is a computational method that determines whether an a priori defined set of genes shows statistically significant, concordant differences between two biological states [24] (e.g., phenotypes) (from the official GSEA website). We used this computational method to analyze the function and potential pathway of signature genes. In order to find out the relationship between the gene set and the function we are interested in, we conducted GSEA analysis based on “C5: GO gene sets” for three groups of GSEs by GSEA software version 4.0.3. The false discovery rate (FDR) < 25% and nominal *P* < 0.05 were regarded as the cut-off criteria.

### 2.3. Survival Analysis

#### 2.3.1. Risk Score Formula Establishment

The clinical information of the original 521 TCGA patients with lung adenocarcinoma was sorted out and 270 cases were screened out, and the patients with lung adenocarcinoma were randomly divided into the training group (*n* = 135) and the testing group (*n* = 135). We further investigated the potential roles in clinical outcomes after screening out the genes. We used a risk-score formula to predict LUAD patients' survival. The risk score formula is as follows: Risk score = (1.43 × expression level of AC069513.4) + (0.81 × expression level of AC003092.1) + (1.64 × expression level of RP11 − 507 K2.3) + (−6.56 × expression level of CTC − 205 M6.2) + (−1.72 × expression level of U91328.21) [25].

#### 2.3.2. Risk Score Formula Validation

To validate the gene risk signature in the internal validation data sets, we calculated the risk score for each patient in the complete TCGA cohort. The patients were then divided into high-risk and low-risk groups based on the corresponding median risk score. The prediction accuracy of this risk model was determined by a time-dependent receiver operating characteristic (ROC) analysis.

#### 2.3.3. Statistical Analysis

Statistical analysis and graphical plotting were conducted by R software. Differences in pathological and molecular characteristics between different groups of patients were compared using chi-squared and Fisher's exact tests. Prognostic factors were assessed by Cox regression analysis and the Kaplan-Meier method. The survival rates were calculated by Kaplan-Meier method curves and compared using the log-rank test. The significance of prognostic factors was evaluated through a multivariate Cox proportional hazard regression, with a *P* value less than 0.05 considered as statistical significant.

Then, the Kaplan-Meier plotter was applied to examine the prognostic value of SHMT2. Kaplan-Meier plotter database (http://kmplot.com/analysis/) is an online analysis tool containing microarray profiles and mRNA-seq data with patients' survival information, including overall survival (OS) and progression-free survival (RFS), summarized from TCGA, Gene Expression Omnibus, and the Cancer Biomedical informatics Grid [26]. Kaplan-Meier plotter database was used to analyze the correlation between SHMT2 expression and survival in LUAD. A log-rank *P* value and the hazard ratio (HR) with confidence intervals of 95% were also calculated.

### 2.4. Signature Gene Online Validation and Analysis

#### 2.4.1. Oncomine Database Analysis

The expression level of SHMT2 in various types of cancers was analyzed in the Oncomine database (https://www.oncomine.org/), especially in lung cancer. Oncomine database is an online cancer database with powerful analytical capabilities for computing gene expression signatures, clusters, and gene-set modules, automatically extracting biological insights from the data [27]. The mRNA expression difference between tumors and normal tissues were analyzed with thresholds as follows: *P* value of 0.01, fold change of 2, gene ranking of all, and the data from mRNA.

#### 2.4.2. GEPIA Database Analysis

The Gene Expression Profiling Interactive Analysis (GEPIA) database (http://gepia.cancer-pku.cn/) is an interactive web for analyzing the expression data of RNA based on 9,736 tumors and 8,587 normal samples from the cancer genome atlas (TCGA) and the GTE projects [28]. We conducted an online survival analysis of the gene SHMT2 on the functional section named as the Survival plot in the GEPIA database. The threshold is determined by the following principles: Gene of SHMT2, Methods of Overall Survival, Group Cutoff of Median, Cutoff-High (%) and Cutoff-Low (%) both are 50, Hazards Ratio (HR) of yes, 95% Confidence Interval of yes, Axis Units of month, and Datasets set as LUAD.

#### 2.4.3. UALCAN Database Analysis

UALCAN database (http://ualcan.path.uab.edu) is a user-friendly and interactive database, providing easy access to RNA-seq and clinical data of 31 cancer types from The Cancer Genome Atlas (TCGA) [29]. We checked the RNA-seq expression of SHMT2 again and further explored the correlation between SHMT2 protein expression and LUAD in this database.

#### 2.4.4. TIMER Database Analysis

The correlations between SHMT2 expression and the abundance of immune infiltrates were explored by the Gene module in the TIMER database (https://cistrome.shinyapps.io/timer/), which is a comprehensive tool established for systematically analyzing immune infiltrates across diverse types of cancer [30]. Meanwhile, we also analyzed the relationship between the expression of SHMT2 and gene markers of tumor-infiltrating immune cells by a correlation module. Besides, the expression level of SHMT2 in various types of cancers was examined in the TIMER database once more.

#### 2.4.5. TISIDB Database Analysis

To further investigate the correlations among SHMT2 expression, lymphocytes, and other immunomodulators, the TISIDB database (http://cis.hku.hk/TISIDB), known as a web portal for tumor and immune system interaction, was applied to analyze. TISIDB integrates multiple heterogeneous data types, including 988 reported immune-related antitumor genes, high-throughput screening techniques, molecular profiling, and para-cancerous multiomics data, as well as various resources for immunological data retrieved from seven public databases [31]. We used the TISIDB database to analyze the link between SHMT2 and immune cell infiltration and to learn the GO function in LUAD.

#### 2.4.6. Human Protein Atlas Analysis

The protein expression of SHMT2 in both LUAD and normal tissues was retrieved from the Human Protein Atlas database (HPA) (https://www.proteinatlas.org/), which is a program with the aim to map all the human proteins in cells, tissues, and organs using an integration of various omics technologies, including antibody-based imaging, mass spectrometry-based proteomics, transcriptomics, and systems biology [32, 33]. In this study, we used the HPA database to analyze the protein expression and performed immunohistochemistry (IHC) analysis of SHMT2 between normal lung tissues and LUAD tissues.

## 3. Results

### 3.1. Identification of Common Differentially Expressed Genes (DEGs)

**Figure 1:**
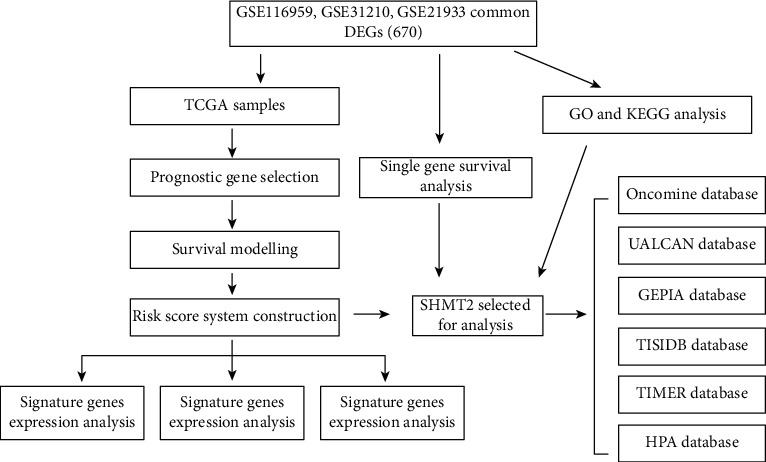
Flowchart for this study. DEGs: differential expression genes; GEPIA: Gene Expression Profiling Interactive Analysis; GO: Gene Ontology; GSEA: gene set enrichment analysis; KEGG: Kyoto Encyclopedia of Genes and Genomes; KM: Kaplan–Meier; TCGA: The Cancer Genome Atlas; TIMER: Tumor Immune Estimation Resource; HPA: the Human Protein Atlas.

**Figure 2:**
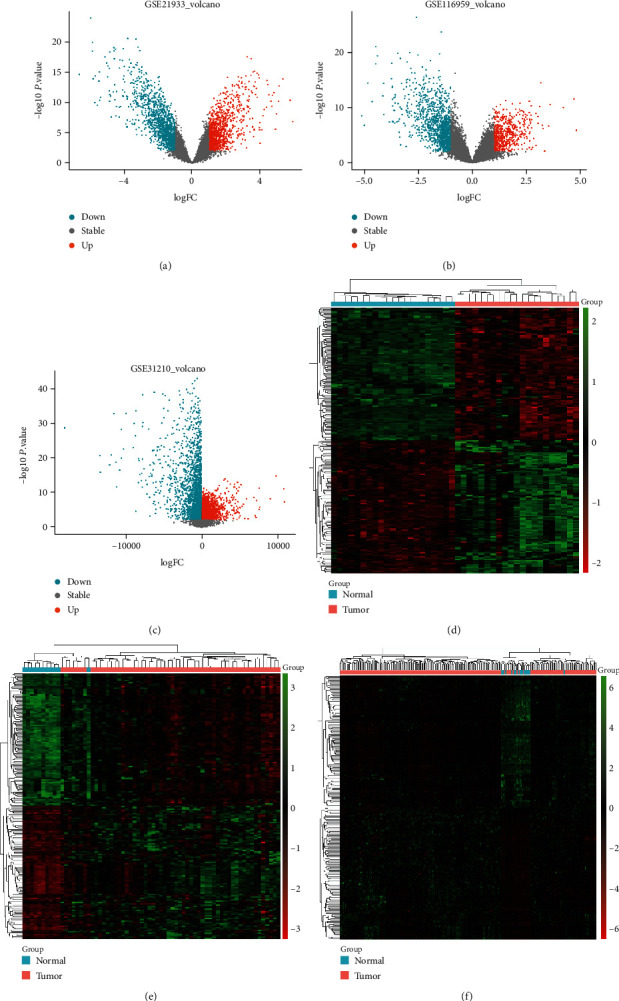
DEGs in three data sets. (a–c) The volcano plots visualize the DEGs in GSE116959, GSE21933, and GSE31210, respectively. The red nodes represent upregulated genes while the blue nodes represent downregulated genes. (d–f) Heatmap of the top 100 DEGs according to the value of ∣logFC | >1 and *P* < 0.01. The green color indicates lower expression and red color indicates high expression.

The flow chart of the study was generalized in Figure 1. After performing difference analysis with dataset collection (GSE116959, GSE21933, and GSE31210) in the GEO database, a total of 577 samples (521 tumor samples and 46 normal samples) from TCGA database were explored. There were 1868 DEGs filtered from the GSE116959 data set, including 624 upregulated and 1244 downregulated genes; 2570 DEGs screened from the GSE21933 data set, including 1193 upregulated and 1377 downregulated genes; and 7220 DEGs selected from the GSE31210 data set, including 4107 upregulated and 3013 downregulated genes. In order to make the results more intuitive, we visualized them. We displayed the DEGs among each data set via volcano plots (Figures 2(a)–2(c)). What is more, cluster analysis of DEGs showed two obvious different distribution patterns between the tumor and normal samples, suggesting crucial roles of DEGs in the occurrence and progression of LUAD (Figures 2(d)–2(f)). Through Venn diagram analysis, 670 common DEGs in the intersection of the three data sets were identified and selected for further analysis.

### 3.2. The Selection of Signature Genes

**Figure 3:**
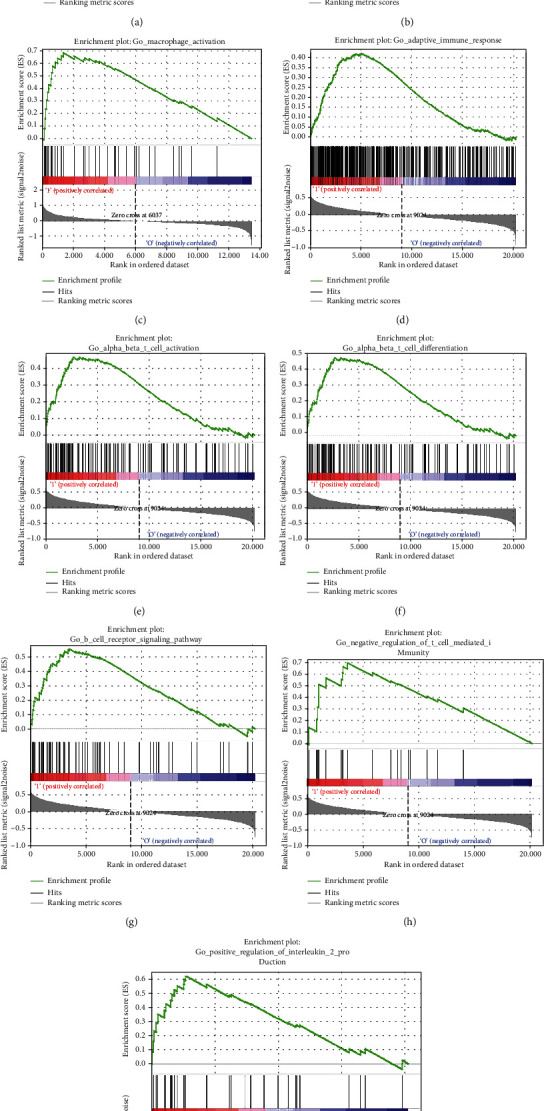
Upregulated gene expression was associated with an immunologic process and was validated by GSEA of GO gene sets analysis of high expression of SHMT2 in GSE21933, GSE31210, and GSE116959. (a) Leukocyte activation involved in inflammatory response, (b) macrophage activation, (c) response to interleukin 6, (d) adaptive immune response, (e) alpha beta T cell activation, (f) alpha beta T cell differentiation, (g) B cell receptor signaling pathway, (h) negative regulation of T cell mediated immunity, and (i) positive regulation of interleukin 2 production.

In order to search for the signature gene, we performed gene set enrichment analysis on GSE21933, GSE31210, and GSE116959. Afterward, with GSEA, we found that there were nine sets of results related to immunity closely, and all of them were highly expressed gene sets. In particular, GSE21933 was associated with macrophage, which is one of our focuses. The information of GSEA results was listed in Figure 3. Next, we selected upregulated genes for further analysis of the differences between genes and enrichment.

**Figure 4:**
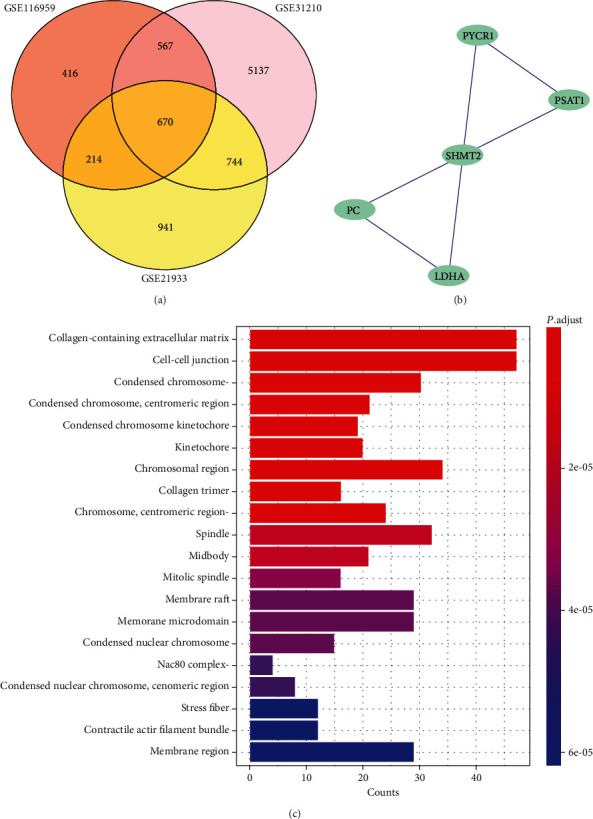
(a) Common DEGs in three data sets. A total of 670 commons in the intersection of three gene sets. (b) Hub gene of common DEGs. There are five hub genes in 670 common genes, including SHMT2, PSAT1, PYCR1, PC, and LDHA. (c) GO analysis of common DEGs.

We conducted GO analysis and KEGG pathway enrichment analysis of DEGs from differential analysis to explore their potential biological functions and pathways associated with LUAD. The results of GO analysis in Figure 4(c) showed that DEGs were significantly related to mitotic nuclear division, cell-substrate adhesion, organelle fission, mitotic sister chromatid segregation, nuclear division, regulation of cell-substrate adhesion, regulation of mitotic nuclear division, microtubule cytoskeleton organization involved in mitosis, chromosome segregation, regulation of chromosome segregation, sister chromatid segregation, mitotic spindle organization, extracellular structure organization, urogenital system development, regulation of nuclear division, DNA-dependent DNA replication, and cell junction assembly, which were essential for the rapid growth of tumors. Additionally, as shown in Figure 4(b), MCODE was used to screen out cluster 15 containing all the upregulated 5 common DEG hub genes (SHMT2, PYCR1, PSA T1, PC, and LDHA) from Cytoscape software, and it was found that SHMT2 gene was located in the specific center of Figure 4(b), indicating that SHMT2 plays an important role in regulating cell behavior. In a function model of TISIDB, we verified the SHMT2 involved in the metabolism of glycine, serine, and threonine, metabolic pathways, carbon metabolism, biosynthesis of amino acids, providing the crucial basis for protein and nucleic acid production for cancer growth and metastasis. Thus, we believe that SHMT2 plays an important role in regulating the growth of LUAD.

### 3.3. High Expression Level of SHMT2 in Tumors

**Figure 5:**
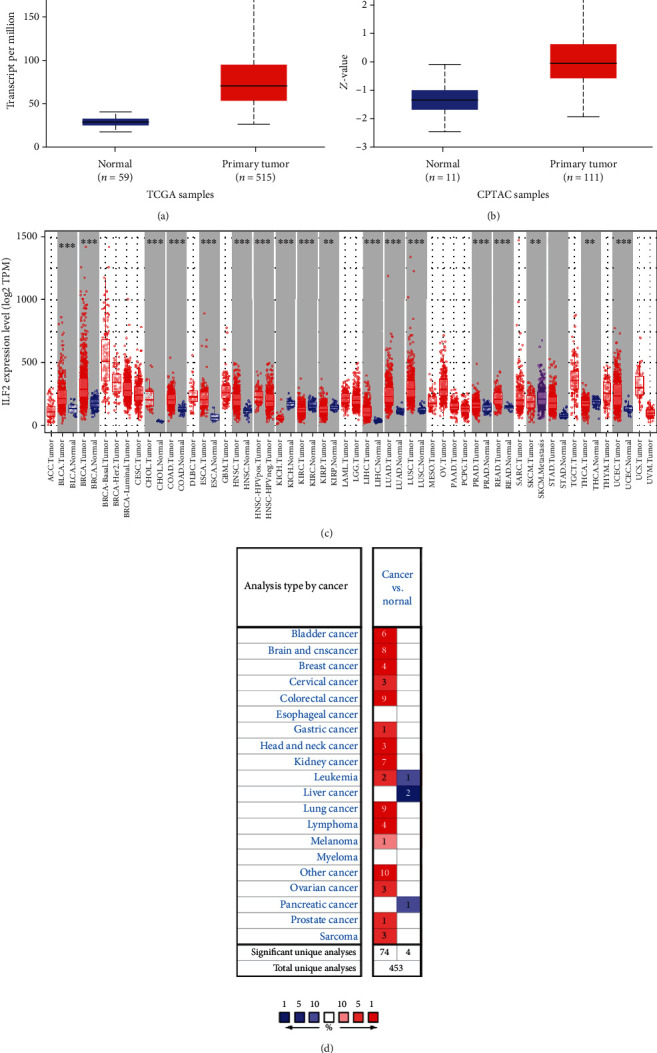
SHMT2 expression level (a) mRNA expression of SHMT2 in LUAD. The mRNA expression of SHMT2 is higher in tumor but lower in normal based on TCGA samples; (b) protein expression of SHMT2 in LUAD. The protein expression of SHMT2 is higher in tumor while lower in normal based on CPTAC samples; (c) the mRNA expression level of SHMT2 in various cancer. Color images are available online. Fold change = 2 and *P* value = 0.01; (d) SHMT2 different expression between tumor and adjacent normal tissue. ^∗^*P* < 0.05, ^∗∗^*P* < 0.01, ^∗∗∗^*P* < 0.001.

**Figure 6:**
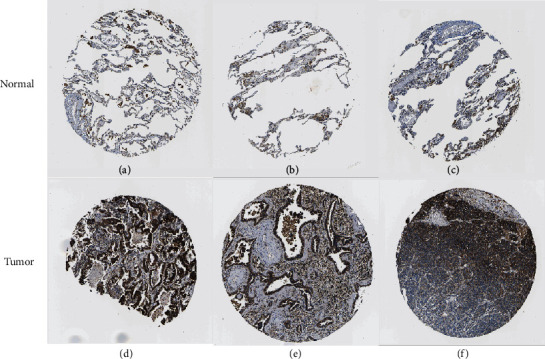
Immunohistochemistry (IHC) of SHMT2 expression in LUAD tissues and corresponding normal tissues based on The Human Protein Atlas (HPA). (a–c) Normal lung (T-28000) tissue and (d–f) lung (T-28000) tumor tissue.

The expression level of SHMT2 in tumor and adjacent normal tissues was verified on the Oncomine database. As shown in Figure 5(d), SHMT2 displayed a higher expression level in bladder cancer, breast cancer, colorectal cancer, kidney cancer, lung cancer, and lymphoma, while the expression level was lower in liver cancer and pancreatic cancer. We also analyzed the mRNA-seq expression data in tumors by UALCAN database and TIMER database (Figures 5(a) and 5(c)). These results consistently showed that SHMT2 displays obviously high expression in LUAD. Besides, we explored the protein expression of SHMT2 between LUAD and normal tissues in UALCAN database (Figure 5(b)) and investigated immunohistochemistry (IHC) on The Human Protein Atlas (HPA) (Figures 6(a)–6(f)). Through the above analysis, we summarized the protein expression of SHMT2 was significantly elevated in tumors which may possess diverse functions in various tumors, especially in LUAD.

### 3.4. Prognostic Value of SHMT2 in LUAD

**Figure 7:**
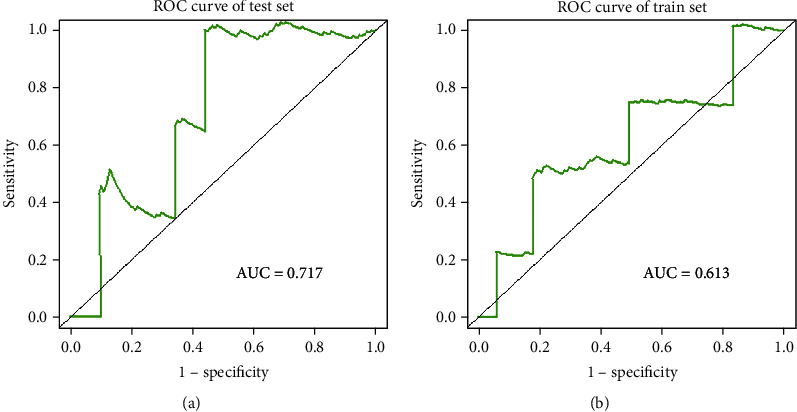
The time-dependent receiver operating characteristic (ROC) analysis. The AUC (area under ROC) score for the training dataset was 0.842, indicating the better performance of survival prediction in the training dataset.

**Figure 8:**
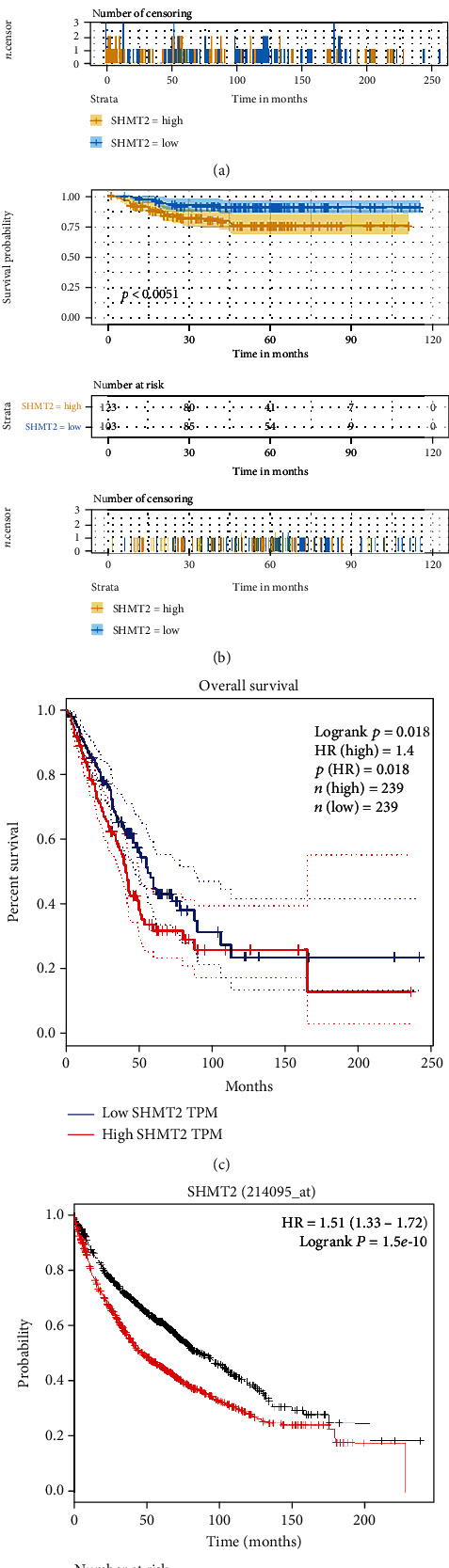
Kaplan-Meier survival analysis. (a) OS (overall survival) of SHMT2 in GSE21933. (b) OS (overall survival) of SHMT2 in GSE31210. The numbers below the figures represent the number of patients at risk in each group. (c and d) Kaplan-Meier survival curves comparing the high and low expression of SHMT2 in LUAD in the Kaplan-Meier plotter database and GEPIA database.

We calculated the area under the curve (AUC) of the receiver operating curve (ROC) to evaluate the discriminative ability of prediction rules. And the AUC score for the training dataset was 0.613 (Figure 7), indicating better survival prediction performance of the training data set. A Kaplan-Meier analysis showed an unfavorable effect on overall patient survival. Multivariate analysis and the Cox proportional hazard regression model uncovered that the expression of SHMT2 is an independent prognostic indicator for patients with LUAD (Figures 8(a) and 8(b)).

We then examined the prognostic value of SHMT2 using the Kaplan-Meier plotter and the Gene Expression Profiling Interactive Analysis (GEPIA) database. We calculated the Cox *P*/log-rank *P* value and hazard ratio with 95% intervals. We set Cox *P*/log-rank *P* = 0.05 as the thresholds. The patients were divided into two groups based on the median level of the SHMT2 expression in each queue. Univariate analysis was carried out to assess the impact of SHMT2 on various cancer survival rates by GEPIA and the Kaplan-Mayer plotter database (Figures 8(c) and 8(d)). The results indicated that the expression level of SHMT2 has a significant effect on the prognosis of LUAD. Moreover, the low level of SHMT2 indicated a longer survival period for patients with LUAD. Given all that, these results suggested that high expression of SHMT2 was related to the poor prognosis of LUAD.

### 3.5. SHMT2 Immune Regulation Molecules

The result of GESA analysis based on 3 datasets in Figure 3 showed that the upregulated gene in GSE21933, GSE31210 was apparently correlated with immune-related biological functions and SHMT2 was proved as a significantly upregulated gene in 3 datasets, suggesting SHMT2 is possibly connected with immune regulation.

**Figure 9:**
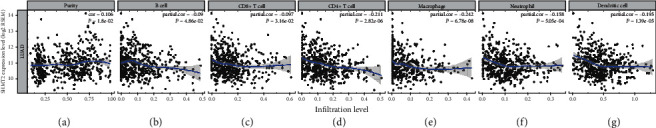
Correlation between SHMT2 expression and immune cell infiltration in LUAD from TCGA sample. Tumor purity, B cell abundance, CD8 + T cells, CD4 + T cells, macrophages, neutrophils, and dendritic cells relative to SHMT2 expression.

In order to explore whether SHMT2 exerts potential biological roles in immune infiltration, we conducted an integrated analysis based on the TIMER database and TISIDB database, analyzing the link between SHMT2 and immune cell infiltration as well as the gene markers of immune cell subtypes in LUAD. The results in Figure 3 suggested high levels of SHMT2 mRNA expression were associated with high immune infiltration in LUAD. SHMT2 mRNA expression level was significantly negatively correlated with infiltrating levels of immune cells, CD4+ T cells (*r* = −0.055, *P* = 2.22*e* − 01), macrophages (*r* = −0.17, *P* = 1.65*e* − 04), and dendritic cells (DCs) (*r* = −0.111, *P* = 1.44*e* − 02) (Figure 9). Besides, Supplemental Table 1 also demonstrated the SHMT2 mRNA expression level had significant correlations with immune cells, TAMs, DCs, CD4+ T cells, neutrophils, Th1, Th2, Thf, and T cell exhaustion in LUAD.

**Figure 10:**
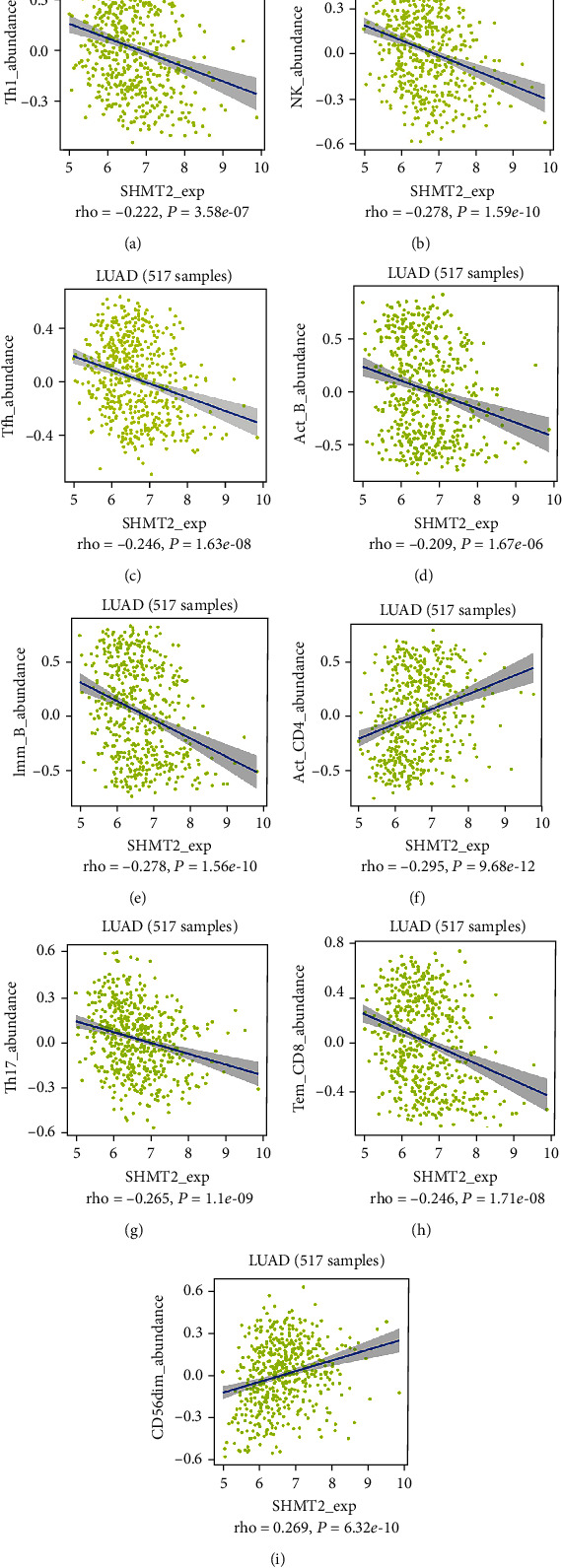
Relations between the abundance of tumor-infiltrating lymphocytes (TILs) and SHMT2 expression, including (a) Type 1 T helper cell, (b) nature killer cell, (c) T follicular helper cell, (d) active B cell, (e) immature B cell, (f) active CD4 T cell, (g) Type 17 T helper cell, (h) Tem CD8, and (i) CD56 dim nature killer cell.

For further investigation, we found the expression of SHMT2 was associated with tumor-infiltrating lymphocytes (TILs), including activated Type 1 T helper cell, nature killer cell, T follicular helper cell, active B cell, immature B cell, active CD4 T cell, Type 17 T helper cell, Tem CD8 cell, and CD56dim nature killer cell (Figures 10(a)–10(i)). Particularly, the *P* value of the abovementioned cells is all less than 0.001. Overall, these results suggested that the SHMT2 and its associated genes were important for immune cell infiltration in the LUAD microenvironment and possibly have a more significant effect on the prognosis of LUAD.

## 4. Discussion

As an important branch of glycolysis and an essential source of one-carbon metabolism [3], serine was essential to support tumor cell proliferation [34]. SHMT, an essential enzyme that catalyze the conversion of serine to glycine, regulates serine metabolism and one-carbon metabolism, to provide important precursors for protein and nucleic acid synthesis for cancer growth and metastasis [3]. SHMT2, a type of SHMT gene found in the human genome, is associated with the prognosis of various tumors [10]. It is reported that that SHMT2 is a key enzyme in the serine/glycine synthesis pathway, catalyzing the transformation of serine into glycine in mammalian mitochondria [12]. SHMT2 may serve as a prognostic factor and as a potential therapeutic target for human gliomas in clinical practice [13, 35]. However, there is still no study on the relationship between SHMT2 and LUAD. Therefore, it is of great significance to analyze the role of SHMT2 in LUAD.

As the most common LUAD, dense lymphocytic infiltrate is one of the most obvious characteristics of LUAD, indicating the immune system exerts an active role in the development and growth of LUAD. In this study, we screened out the key gene SHMT2 through difference analysis, functional enrichment analysis, and survival analysis based on the GEO database and TCGA database. Next, we used the Oncomine database and TIMER database to compare the expression level of SHMT2 among different cancers and verify its increased expression level in LUAD. Univariate analyses of this study were carried out to evaluate the effect of SHMT2 expression on the survival rates in LUAD via the R software and Kaplan-Meier plotter database. The high expression level of SHMT2 had a more significant effect on the prognosis of LUAD patients. After screening tumor prognosis related to SHMT2, the relationship between SHMT2 and immune infiltration levels in different tumors was investigated in the TIMER database and TISIDB database. The levels of infiltration of immune cells in LUAD were performed on the TIMER database, revealing that SHMT2 is obviously related to the immune filtration in this cancer.

Besides, multivariate analysis and the Cox proportional hazard regression model validated that SHMT2 could be an independent prognostic factor of patients with LUAD. The expression level of SHMT2 also had a significantly negative correlation with tumor-infiltrating lymphocytes like immature B cell, active CD4 T cell, Th17, CD56dim nature killer cell (all Cor > 0.2; *P* < 0.01). Additionally, the results of correlation between SHMT2 and gene markers of immune cells showed that the SHMT2 was closely related with T cells (CD8 + T cells, Th1 cells, Th2 cells, Thf cell, general T cells, and exhausted T cells), TAM, NK cells, and DCs (Supplemental Table 1). Tumor-infiltrating lymphocytes (TILs), including T cells and B cells, are another important component of immune cells that exhibit antitumoral functions, especially CD8 and CD4 T cells. Some studies revealed that Th1 cells were associated with prolonged survival. SHMT2 regulating immune infiltration may be involved in these immune cells, especially T cell receptor interaction. The analysis mentioned above suggested that SHMT2 could serve as a potential overall prognostic marker for patient survival, improving the survival and prognosis of LUAD; SHMT2 may also play an important role in the microenvironment of LUAD via regulating tumor infiltration of immune cells.

At present, according to the known research results, the high expression of SHMT2 could be detected in different types of cancers, as reported, playing pivotal roles in =migration and invasion. Knocking out SHMT2 in hepatocellular cancer cell lines was validated that reduces cell growth and tumorigenicity in vitro and vivo. Gene set enrichment analysis revealed that SHMT2 had a strong correlation with cancer invasion and poor survival among breast cancer patients. Besides, SHMT2 also was reported to control inflammatory cytokine signaling via its interaction with the BRISC deubiquitylase (DUB) and its important catalyst [36]. And SHMT2 impaired T cell survival in culture and antigen-specific T cell abundance in vivo [37]. Overall, these studies provide evidence that SHMT2 participated in different diseases via immune mechanisms.

## 5. Conclusion

In this study, we showed SHMT2 as an independent prognostic factor and found its high expression was associated with poor prognosis of LUAD. And further analysis conjectured that SHMT2 may mediate the immune cell infiltration via regulation of macrophages and T cell in the LUAD microenvironment. Although there are some shortcomings in this study, such as our lack of experimental verification, we also demonstrate some highlights, which deserve more attention. We take full advantage of available public online datasets to verify our conjecture. However, further exploration and research to study the specific mechanism are also required. We hope this article can contribute to the following research.

## Data Availability

The data that support the findings of this study are available from the corresponding author upon reasonable request.

## Supplementary Materials

Supplementary Materials Supplementary Table 1: correlation analysis between SHMT2 and related gene markers of immune cells.
