# Supplementary material for: Identification of SHMT2 as a Potential Prognostic Biomarker and Correlating with Immune Infiltrates in Lung Adenocarcinoma
Source: J Immunol Res. 2021 Apr 8;2021:6647122. doi: 10.1155/2021/6647122 (PMC8049788; doi:10.1155/2021/6647122)
Supplement: Supplementary Materials — Supplementary Table 1: correlation analysis between SHMT2 and related gene markers of immune cells. [file 6647122.f1.docx]

Supplementary table 1

Correlation analysis between SHMT2 and related gene markers of immune cells.

*P<0.05, **P<0.01, ***P<0.001

| **Description** | **Gene marker** | **SHMT2** | | | |  | **Description** | **Gene marker** | **SHMT2** | | | |
| --- | --- | --- | --- | --- | --- | --- | --- | --- | --- | --- | --- | --- |
|  |  | **None** | | **Purity** | |  |  |  | **None** | | **Purity** | |
|  |  | **cor** | **p** | **cor** | **p** |  |  |  | **cor** | **p** | **cor** | **p** |
| **TAM** | **VSIG4** | **-0.0859** | ****** | **-0.05576** | **0.079945** |  | **B cell** | **CD40** | **-0.13984** | ****** | **-0.10275** | ***** |
|  | **CSF1R** | **-0.11735** | ****** | **-0.07542** | **0.094366** |  |  | **CD80** | **-0.09015** | ***** | **-0.05303** | **0.239835** |
|  | **FCGR2A** | **-0.09113** | ***** | **-0.05383** | **0.232868** |  |  | **CXCR4** | **-0.09868** | ***** | **-0.07199** | **0.110367** |
|  | **FCER2** | **-0.19299** | ******* | **-0.15948** | ******* |  |  | **CXCR5** | **-0.11635** | ****** | **-0.07293** | **0.105789** |
| **Treg** | **FOXP3** | **0.024662** | **0.576457** | **0.085218** | **0.058653** |  |  | **TLR4** | **-0.13316** | ****** | **-0.1059** | ***** |
|  | **STAT5B** | **-0.06034** | **0.171461** | **-0.05006** | **0.267301** |  | **Dendritic celll** | **HLA-DPA1** | **-0.25847** | ******* | **-0.23163** | ******* |
|  | **TGFB1** | **-0.19353** | ******* | **-0.16703** | ******* |  |  | **CCR7** | **-0.18066** | ******* | **-0.05576** | **0.216479** |
|  | **CCR8** | **-0.00152** | **0.972596** | **0.049541** | **0.27226** |  |  | **ITGAM** | **-0.17099** | ******* | **-0.05383** | **0.232868** |
| **Th1** | **STAT4** | **-0.18081** | ******* | **-0.15209** | ******* |  |  | **CD59** | **-0.39605** | ******* | **0.085218** | **0.058653** |
|  | **CD4** | **-0.16932** | ******* | **-0.13212** | ****** |  |  | **HLA-DPB1** | **-0.30492** | ******* | **-0.28417** | ******* |
|  | **STAT1** | **0.161045** | ******* | **0.207551** | ******* |  |  | **HLA-DQB1** | **-0.16634** | ******* | **-0.136** | ****** |
|  | **IFNG** | **0.120743** | ****** | **0.165272** | ******* |  |  | **HLA-DRA** | **-0.2821** | ******* | **-0.26451** | ******* |
| **Th2** | **GATA3** | **-0.08165** | **0.064121** | **-0.03198** | **0.478618** |  |  | **ITGAX** | **-0.09346** | ***** | **-0.05407** | **0.230776** |
|  | **CXCR4** | **-0.09868** | ****** | **-0.07199** | ***** |  |  | **CD1C** | **-0.3915** | ******* | **-0.37063** | ******* |
|  | **CCR4** | **-0.17256** | ******* | **-0.1376** | ****** |  |  | **THBD** | **-0.1806** | ******* | **-0.16764** | ******* |
| **Tfh** | **IL21** | **0.134901** | ****** | **0.174578** | ******* |  | **Natural killer cell** | **KIR2DL1** | **0.071633** | **0.104432** | **0.082454** | **0.067362** |
|  | **BCL6** | **-0.12226** | ****** | **-0.11344** | ***** |  |  | **KIR2DL3** | **0.094744** | ***** | **0.115068** | ***** |
| **T Cell exhaustion** | **PDCD1** | **0.103873** | ***** | **0.167267** | ******* |  |  | **KIR2DL4** | **0.254623** | ******* | **0.284648** | ******* |
|  | **CTLA4** | **-0.00659** | **0.881405** | **0.054575** | **0.22644** |  |  | **KIR3DL1** | **0.05615** | **0.203316** | **0.080966** | **0.072474** |
|  | **LAG3** | **0.157443** | ******* | **0.205703** | ******* |  |  | **KIR3DL2** | **0.120751** | ****** | **0.14666** | ****** |
|  | **GZMB** | **0.262616** | ******* | **0.323581** | ******* |  |  | **KIR3DL3** | **0.171332** | ******* | **0.193336** | ******* |
| **Neutrophli** | **CCR7** | **-0.18066** | ******* | **-0.14587** | ******* |  | **M1 Macrophage** | **IRF5** | **-0.15333** | ******* | **-0.13549** | ****** |
|  | **ITGAM** | **-0.17099** | ******* | **-0.13038** | ******* |  |  |  |  |  |  |  |
|  | **CD59** | **-0.39605** | ******* | **-0.38037** | ******* |  |  |  |  |  |  |  |
|  | **CCR7** | **-0.18066** | ******* | **-0.14587** | ****** |  |  |  |  |  |  |  |
